# Supplementary material for: Using genetics, genomics, and transcriptomics to identify therapeutic targets in juvenile idiopathic arthritis
Source: HGG Adv. 2025 Mar 13;6(2):100424. doi: 10.1016/j.xhgg.2025.100424 (PMC11994403; doi:10.1016/j.xhgg.2025.100424)
Supplement: Document S1. Table S2 [file mmc1.pdf]

HGGA, Volume *6*

Supplemental information

Using genetics, genomics, and transcriptomics to identify therapeutic targets in juvenile idiopathic arthritis

Evan Tarbell and James N. Jarvis

Supplemental Table 2. Clinical Trials of putative gene targets

ClinicalTrials.gov ID
NCT00034203
NCT00276250
NCT00287118
NCT00669214
NCT00312026
NCT00302445
NCT00096980
NCT00336973
NCT00249808
NCT00737763
NCT00501709
NCT00051662
NCT00000278
NCT02101008
NCT03950830
NCT00312819
NCT00435435
NCT02735577

Clinical trial identification numbers for drugs against one of the putative target genes for JIA.
