# Supplementary material for: Using genetics, genomics, and transcriptomics to identify therapeutic targets in juvenile idiopathic arthritis
Source: HGG Adv. 2025 Mar 13;6(2):100424. doi: 10.1016/j.xhgg.2025.100424 (PMC11994403; doi:10.1016/j.xhgg.2025.100424)
Supplement: Document S2. Article plus supplemental information [file mmc3.pdf]

HGG Advances

ARTICLE

Using genetics, genomics, and transcriptomics to identify therapeutic targets in juvenile idiopathic arthritis

Evan Tarbell[1,*] and James N. Jarvis[2,3,*]

Summary

Despite progress in improving outcomes for oligoarticular and polyarticular juvenile idiopathic arthritis (JIA), the field still faces considerable challenges. More than half of adults who have had JIA continue to have active disease and have developed functional limitations. Medication side effects are common and intrusive. Thus, the field continues to search for therapeutic agents that target specific aspects of disease pathobiology and will be accompanied by fewer and less intrusive side effects. We identified 28 candidate target genes that were associated with JIA according to Open Targets Genetics and were also differentially expressed in the CD4+ T cells of children with active JIA (when compared to healthy control subjects). Of the 28 candidates, the strongest new target to emerge was homeodomain-interacting protein kinase 1 (HIPK1), which suppresses T cell activation and is within the *PTPN22* locus tagged by rs6679677. This locus includes an enhancer element that contacts the HIPK1 promoter, and HIPK1 shows decreased expression in JIA CD4+ T cells when compared to controls. Gene Ontology terms associated with HIPK1 were overrepresented among the differentially expressed genes between JIA and controls, and PML, a known coregulator of HIPK1, showed a similar suppressed gene expression profile. Two downstream transcription factors of HIPK1, TP53 and GATA4, showed enriched binding patterns near the promoters of JIA up-regulated genes. Taken together, these data suggest a pathogenic role for HIPK1 in JIA and make it a prime candidate for therapeutic modulation.

Introduction

Juvenile idiopathic arthritis (JIA) is a chronic condition characterized by chronic inflammation of the synovium and one of the most common chronic conditions affecting children in the USA.[1,2] Considerable progress has been made in treating children with JIA over the past 20 years, particularly with the introduction of biological agents.[3] However, the field still faces important challenges. Despite progress in improving functional outcomes, these outcomes have come at a cost, as most children experience significant and intrusive medication side effects from the initiation of treatment.[4] Furthermore, we face additional serious challenges: (1) we have had limited success in getting children off medication,[5] meaning that children with JIA have prolonged exposure to potent immunosuppressive medications, and (2) half of all adults diagnosed with JIA continue to have active disease and significant functional limitations, which results in exposure to these medications, and their toxicities/side effects, that continue into adulthood.[6]

In the past 5 years, investigators have assessed the potential of using genetic, genomic, and transcriptomic data to guide the development of new therapies or to repurpose known agents.[7,8] The underlying premise of these investigations is that these datasets, used together, are likely to identify features of pathobiology that are not discernable from any single measurement/assessment. Furthermore, by elucidating genetic risk and the immunologic pathways influenced by risk-inducing variants, there is reason to believe that personalized approaches to therapy can be provided to individual patients.

While genetic risk is likely to be exerted on multiple cell types,[9] it has long been known that CD4+ T cells are critical drivers of the immunopathological events in JIA.[10,11] Distinct abnormalities in peripheral blood T cells have been described,[12] and there is ongoing interest in these cells as therapeutic targets.[13]

In this paper, we demonstrate the feasibility of using the known JIA genetic risk loci, three-dimensional (3D) chromatin data, and RNA sequencing (RNA-seq) to identify candidate therapeutic targets. We identified twenty-eight candidate targets, one of which, homeodomain-interacting protein kinase 1 (HIPK1), is novel and represents a potentially druggable pathway.

Material and methods

Patients and control subjects

Patient data (RNA-seq, chromatin immunoprecipitation [ChIP]-seq, HiChIP, and ATAC-seq) used for these studies were previously reported in an earlier paper from our group.[10] We repeat some details on the patient characteristics here. Patients fit criteria for polyarticular-onset, rheumatoid-factor-negative JIA as established by the International League Against Rheumatism (ILAR).[14] We used the Wallace criteria[15–17] to characterize children as having active disease on therapy (ADT) or clinical remission on medication (CRM). Patients with JIA were all being treated with combinations

[1]Enhanced Pharmacodynamics, LLC, Buffalo, NY, USA; [2]Department of Pediatrics, University of Washington School of Medicine, Seattle, WA, USA
[3]Lead contact
*Correspondence: evantarbell@gmail.com (E.T.), jamesjar@uw.edu (J.N.J.)
https://doi.org/10.1016/j.xhgg.2025.100424.
© 2025 The Author(s). Published by Elsevier Inc. on behalf of American Society of Human Genetics.
This is an open access article under the CC BY license (http://creativecommons.org/licenses/by/4.0/).

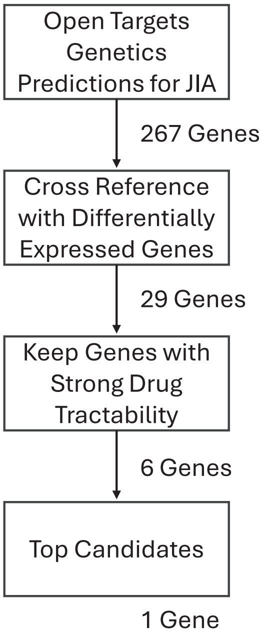

Figure 1. Workflow to identify candidate drug targets for JIA
Flowchart showing the steps to identify a candidate drug target for JIA, along with the number of genes identified at each step.

of methotrexate and the tumor necrosis factor (TNF) inhibitor etanercept. Samples were obtained from children classified as ADT and were obtained 6 weeks from the onset of therapy. Samples obtained from children with CRM were obtained when CRM status was confirmed. This was typically 12–15 months after the initial diagnosis.

Healthy control (HC) children were recruited from the Hodge General Pediatrics Clinic of the University at Buffalo Jacobs School of Medicine and Biomedical Sciences. Exclusion criteria included a fever $\geq$ 38°C within the previous 48 h, the presence of another autoimmune disease (e.g., type 1 diabetes), and treatment with systemic glucocorticoids or antibiotics.

Laboratory data

Data from RNA-seq and ChIP-seq/HiChIP for CTCF in purified CD4+ T cells are available on the Gene Expression Omnibus under accession number GEO: GSE164215, uploaded in January 2021 and updated in April 2021. These data were used for the analyses described below.

The STRINGDB database[18] for protein-protein interactions was utilized to identify the interacting partners to HIPK1. Each member of the interaction network was used as a search term for FactorBook,[19] a repository of publicly available transcription factor binding data. Two of the interacting proteins were identified as transcription factors, TP53 and GATA4. FactorBook listed two TP53 ChIP-seq datasets (accessions ENCFF849LSO in HepG2 cells and ENCFF699UTZ in the A549 cell line) and one for GATA4 (accession ENCFF154VTV in HepG2 cells), which were downloaded.

Processing RNA-seq data

All RNA-seq data analyses were performed on the Galaxy bioinformatics online platform.[20] Raw RNA-seq FASTQ files were downloaded from the SRA archive[21] using the fastq-dump tool.[22] The paired-end FASTQ files were aligned to the hg38 genome using RNA-STAR,[23] using the GENCODE[24] v.44 GTF file as the gene model for splice junctions and with the "GeneCounts" option set to create a table of read counts per gene. All other parameter settings were left on their Galaxy defaults. These count table files were used as the input to the edgeR differential expression analysis tool,[25] which was run without low-count genes, defined as having a cpm value below 1.0 in less than 10 samples, and with all other settings at their default values, including the false discovery rate (FDR) threshold for defining differentially expressed genes (DEGs), which was set to 0.05. DEGs were used for GO term analysis using GOrilla.[26] GOrilla was run using the two gene set option, with the up-regulated or down-regulated genes as the input and the total list of expressed genes as the background set.

Determining regulatory potential of transcription factors for DEGs

BETA[27] was used to calculate the regulatory potential of the HIPK1-associated transcription factors for the DEGs in JIA T cells.

The regulatory potential, which is a gene's likelihood of being regulated by a factor, is estimated for each gene. The regulatory potential is calculated as

$$s_g = \sum_{i=1}^{k} e^{-(0.5+4\Delta i)}.$$

All binding sites (k) near the transcription start site (TSS) of the gene (g) within a user-specified range (100 kb as default) are considered. Δ is the exact distance between a binding site and the TSS proportional to 100 kb (Δ = 0.1 means the exact distance = 10 kb). BETA then generates a cumulative distribution function of the gene groups and uses a one-tailed Kolmogorov-Smirnov test to determine whether the up-regulated and down-regulated groups differ significantly from the non-differential group.

Results

The following approach was employed in order to identify the pathogenic genes that are controlled by non-coding JIA-associated single-nucleotide polymorphisms (SNPs) and then determine which of those represented viable putative drug targets (Figure 1). First, the Open Targets Genetics (OTG) platform[28] was used to link potential pathogenic genes to JIA-associated genetic variants. We find that there were a total of 267 predictions made by OTG, of which 30 were targets of known clinical-stage drugs (Table S1). Those genes whose link to JIA was predicted by OTG were then cross-referenced with genes that were determined to be differentially expressed in CD4+ T cells between patients with active JIA and HC subjects, originally published by Tarbell et al.[10] A total of 29 genes met these criteria (Table 1). With the focus on novel drug targets, genes were then excluded if there were known clinical-stage drugs that targeted them. Four of the 29 genes initially identified met these criteria: *ALDH2*, which has been targeted for alcohol disorders and tested for some cancers (Table S2); *CHRNB2*, a target of nicotine; *ITGAL* (CD11a) was targeted for psoriasis, arthritis, and diabetes

Table 1. JIA-associated gene targets with differential expression

JIA-associated SNP rsID	Putative gene target	Log2(fold change)	FDR value	Known clinical-stage drugs
rs12430303	AKAP11	0.329	0.026	no
rs6434390	ASNSD1	−0.410	0.021	no
rs4616297	ATXN2L	0.431	0.001	no
rs4616297	BCL7C	−0.488	0.017	no
rs2481065	CKS1B	−0.851	0.010	no
rs2481065	DENND4B	0.345	0.014	no
rs2481065	DPM3	−0.845	0.015	no
rs4766578	ERP29	−0.555	0.001	no
rs4616297	FBRS	0.406	0.005	no
rs4869314	GLRX	−0.915	0.001	no
rs2481065	HAX1	−0.638	0.001	no
rs4766578	HECTD4	0.354	0.020	no
rs6679677	HIPK1	0.351	0.016	no
rs4616297	ITGAX	1.419	0.001	no
rs2481065	JTB	−0.481	0.005	no
rs9960807	LDLRAD4	0.428	0.011	no
rs79815064	NBEAL2	0.839	0.001	no
rs6679677	PPM1J	1.830	0.002	no
rs9960807	PSMG2	−0.496	0.010	no
rs9960807	PTPN2	−0.386	0.024	no
rs4616297	SETD1A	0.347	0.031	no
rs4766578	SH2B3	0.647	0.001	no
rs4616297	SRCAP	0.463	0.001	no
rs4616297	TUFM	−0.319	0.036	no
rs4766578	VPS29	−0.577	0.001	no
rs4766578	ALDH2	1.002	0.001	yes
rs2481065	CHRNB2	−1.637	0.031	yes
rs4616297	ITGAL	0.428	0.001	yes
rs4616297	ITGAM	1.090	0.001	yes

List of genes that are associated with JIA, as determined by Open Targets Genetics, and differentially expressed in the CD4+ T cells of patients with JIA versus healthy control subjects. Listed are the fold change and corrected p value of the expression differences and whether there are any clinical-stage or approved drugs that target the gene.

(Table S1); and *ITGAM* (CD11b), which is frequently, although unsuccessfully, targeted for cancers.[29] We find that although our approach (OTG plus DEGs) has a modestly higher incidence of known drug targets (14.3%) as opposed to OTG only (11.2%), the difference is not statistically significant (Fisher's exact test $p = 0.5$).

The remaining 25 identified genes were then assessed for drug tractability using the Open Targets Platform, which includes tractability data that identify key details for small molecules, antibodies, and other modalities. For small molecules, assessments include whether the target has been co-crystallized with a small molecule, whether there is a high-quality ligand, whether there is a high-quality binding pocket, whether there is a med-quality pocket, or if the target is part of a druggable family, as per Finan et al.'s Druggable Genome pipeline.[30] For antibodies, the assessments include whether there is high confidence that the subcellular location of the target is either plasma membrane, extracellular region/matrix, or secretion or medium confidence that the subcellular location of the target is either plasma membrane, extracellular region/matrix, or secretion; whether the target has a predicted signal peptide or transmembrane regions and not destined to organelles; or whether there is high confidence that the target is located in the plasma membrane. Overall, 6 of the 25 candidate genes/gene products met at least one of these

Table 2. JIA-associated genes with strong drug tractability

JIA-associated SNP rsID	Putative gene target	Log2(fold change)	FDR value	Subcellular localization
rs12430303	AKAP11	0.329	0.026	cytosol, nucleoli, plasma membrane
rs2481065	CKS1B	−0.851	0.010	cytosol, mitochondria
rs4869314	GLRX	−0.915	0.001	cytosol, plasma membrane
rs6679677	HIPK1	0.351	0.016	cytosol, nucleoplasm
rs9960807	PTPN2	−0.386	0.024	nucleoplasm
rs4766578	VPS29	−0.577	0.001	cytosol, vesicles

List of genes that are associated with JIA, as determined by Open Targets Genetics, are differentially expressed in the CD4+ T cells of patients with JIA versus healthy control subjects, and had strong drug tractability for either small molecules or antibodies. Listed are the fold change and corrected *p* value of the expression differences and their subcellular location.

criteria for either small-molecule or antibody tractability (Table 2). Of these top 6 candidates, the most promising one was HIPK1, a putative effector gene of the JIA-associated SNP rs6679677. The OTG platform was used to identify 10 putative effector genes of the rs6679677 SNP (Table 3). The highest-scoring target gene from OTG was *PTPN22*, followed by the nearest gene in linear genomic space, *PHTF1*, and HIPK1 rounding out the top 5. Of note is that *HIPK1*, although it had the fifth overall score assessed by OTG, had the third-highest rank in expression quantitative trait locus (eQTL) colocalization scores and chromatin interaction scores, components of the OTG model that use experimental data. To further prioritize the putative targets from OTG, publicly available transcriptomic and genomic data in the CD4+ T cells of patients with JIA and age-matched HC subjects, originally published by Tarbell et al.,[10] were downloaded from the SRA data repository and processed (see material and methods). Transcriptomic data, in the form of RNA-seq, were pooled and averaged across the subjects in each disease group, as were genomic data in the form of ATAC-seq, CTCF ChIP-seq, and HiChIP, and graphically displayed in a genome browser, centered on the rs6679677 SNP (Figure 2). As shown in the genome browser screenshot, there are several regulatory regions in the vicinity of the SNP, denoted by spikes in the ATAC-seq signals. Those regulatory elements near the rs6679677 SNP are in close physical proximity to numerous distal regulatory elements, as shown with the HiChIP loops. Finally, many genes in the vicinity of the rs6679677 SNP and linked to it via chromatin loops show robust expression, as shown in RNA-seq signal tracks. Differential expression analysis between the patients and healthy subjects revealed three genes that both met the expression criteria and were physically linked to the rs6679677 SNP, only one of which, *HIPK1*, was also linked to the SNP via OTG. The normalized gene expression values between disease states for *HIPK1*, showing lower expression in individuals with active disease compared to HC subjects, are shown in Figure 3A. As shown in Tables 1 and 2, HIPK1 had a 1.28-fold decrease in T cell expression in individuals with active disease relative to HC subjects, with an adjusted *p* value of 0.016. To ensure that the expression differences we observed were disease associated rather than the result of treatment administration, we

Table 3. Predicted gene targets of the rs6679677 JIA-associated SNP

Gene	Overall L2G score	Partial L2G scores: Variant pathogenicity	Distance	QTL coloc	Chromatin interaction	Distance to locus
PTPN22	0.352	0.718	0.198	0.287	0.219	110,567
PHTF1	0.236	0.049	0.266	0.032	0.034	1,697
RSBN1	0.092	0.049	0.194	0.035	0.038	51,290
DCLRE1B	0.035	0.064	0.030	0.277	0.155	143,433
HIPK1	0.030	0.064	0.029	0.243	0.155	168,138
BCL2L15	0.027	0.050	0.035	0.035	0.037	126,395
AP4B1	0.018	0.060	0.020	0.038	0.044	144,015
OLFML3	0.017	0.061	0.018	0.130	0.142	218,205
MAGI3	0.012	0.049	0.013	0.016	0.028	370,671
SYT6	0.010	0.049	0.009	0.032	0.034	392,694

Putative target genes of the rs6679677 SNP, as defined by Open Target Genetics. Listed is the overall lead to gene score from Open Targets for each gene associated with the SNP, along with the partial L2G scores from the Open Targets model.

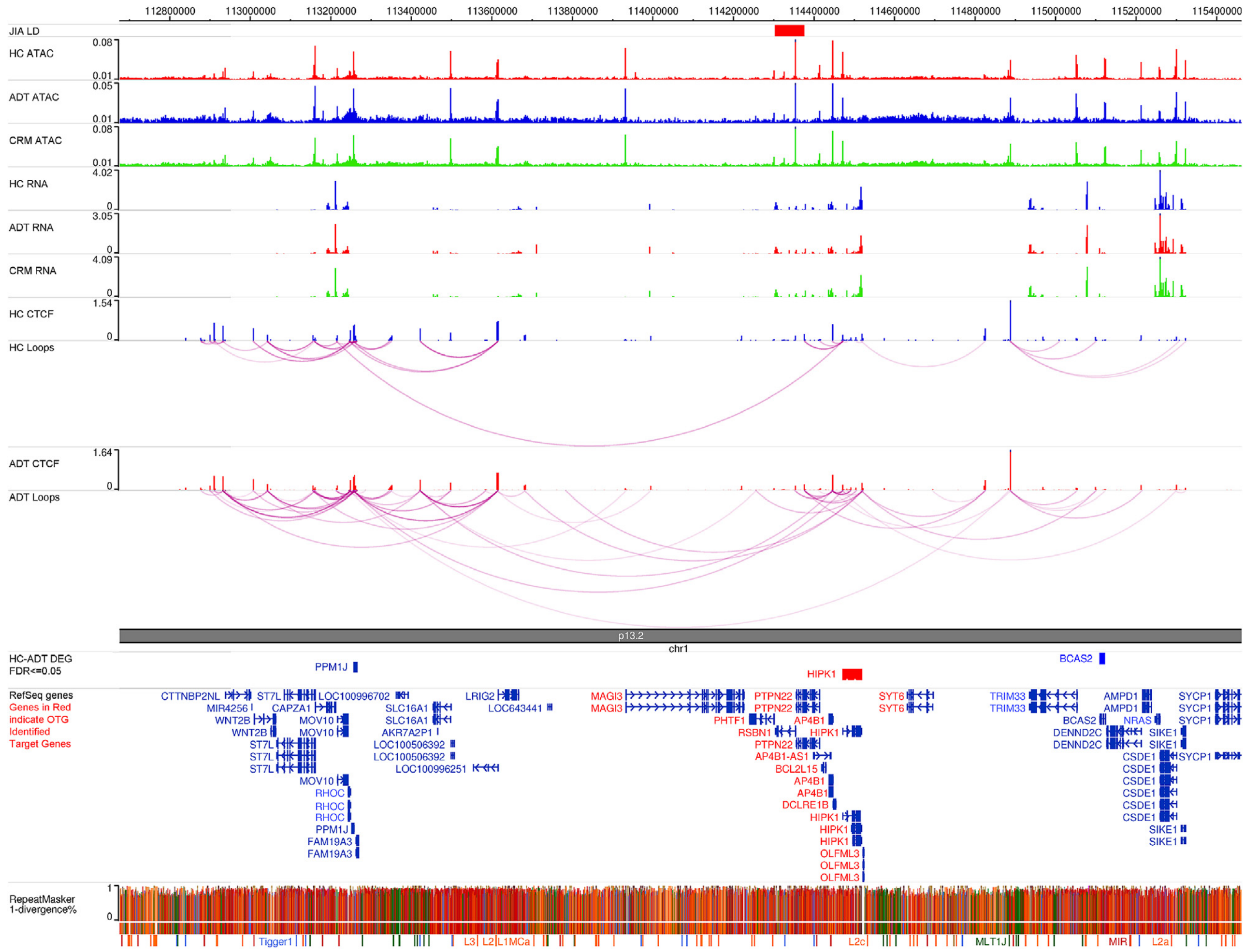

Figure 2. Genome browser snapshot of the rs6679677 JIA-associated SNP
Genome browser snapshot shows data generated in T cells of patients with JIA (active disease under treatment [ADT] and clinical remission on medication [CRM]) and matched healthy control (HC) subjects. Pooled ATAC-seq, RNA-seq, CTCF ChIP-seq, and CTCF HiChIP data are shown, as generated in Tarbell et al.[10] Genes in red indicate genes identified by Open Targets Genetics as target genes of the rs6679677 SNP.

performed differential gene expression analysis on CD4+ T cells from patients in clinical remission with ongoing treatment versus individuals with active disease and HC subjects and found that HIPK1 had a 1.25-fold decrease in T cell expression in active disease relative to clinical remission, with an adjusted p value of 0.035. There was no statistical difference in HIPK1 expression between HC subjects and patients in clinical remission (edgeR DEG analysis, fold change = 1.03, adjusted p value = 1.0). Finally, we find that HIPK1 is predicted to be haploinsufficient and considered to be very intolerant to loss-of-function mutations, according to gnomAD,[31] further demonstrating the deleterious effect of HIPK1 reduction. Overall, these data suggest that the suppression of *HIPK1* in CD4+ T cells may be mediated by the rs6679677 SNP, which alters the regulatory activity of distal elements that influence *HIPK1* expression via physical proximity.

To elucidate the downstream events of *HIPK1* suppression in CD4 T cells and their role in the pathogenesis of JIA, additional analysis was performed. GO term analysis of the DEGs between active disease and healthy controls was conducted using a statistical overrepresentation test to identify enriched terms compared to a random expectation. From this analysis, we ranked and selected the top 15 most enriched GO terms, rather than all terms below a predefined threshold, to focus on the biological processes most relevant to the study. Among these top terms, several were directly associated with HIPK1, underscoring its involvement in key pathways implicated in JIA pathogenesis. By highlighting these specific terms, we aimed to illustrate HIPK1's role in critical biological functions and emphasize its potential as a therapeutic target in JIA (Table 4).

Understanding the role of different genes in the gene regulatory network of *HIPK1* may shed light on the role *HIPK1* plays in JIA. To that end, the gene regulatory network for *HIPK1* was determined using StringDB, a repository of protein-binding data (Figure 3B). The PML nuclear body scaffold protein, a binding partner of and coregulator with HIPK1, was found to be differentially

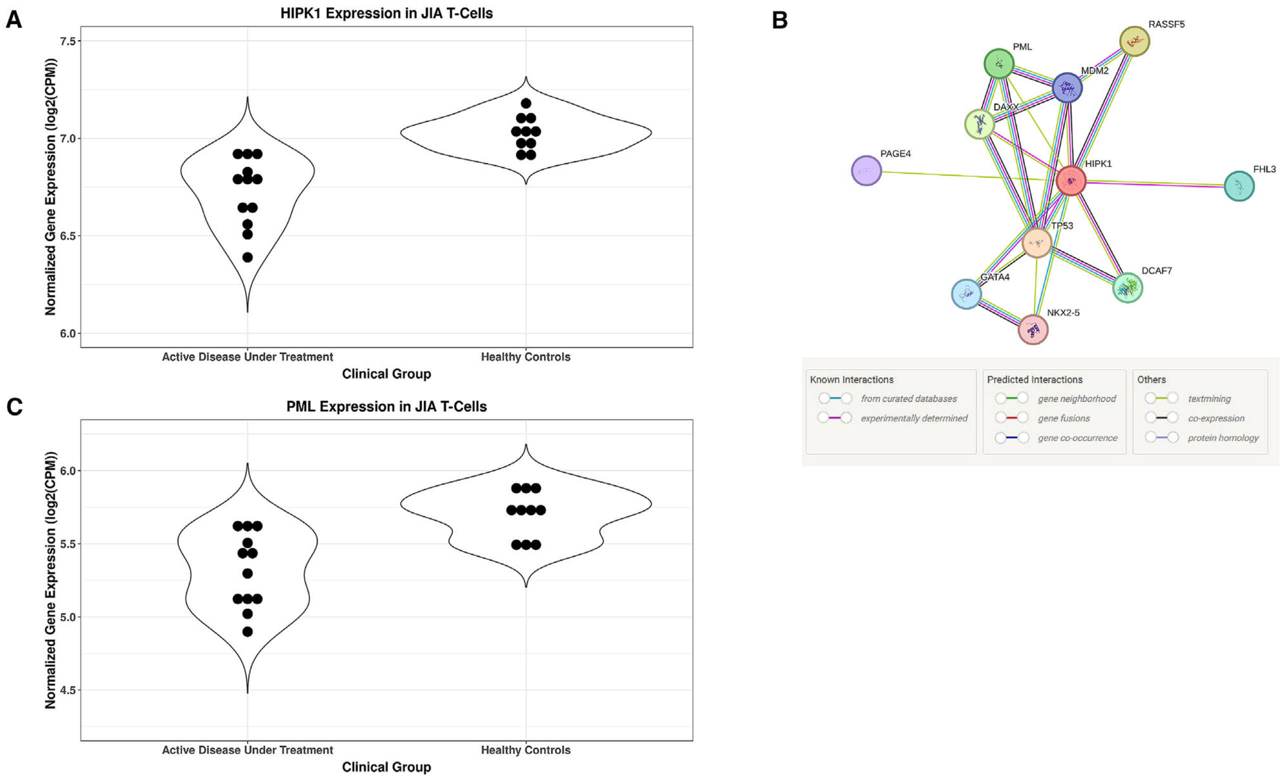

Figure 3. HIPK1 and its gene network partners show disease-associated expression patterns
(A) Violin plots of HIPK1 normalized gene expression, measured as log2(counts per million), for healthy control subjects and patients with active disease and under treatment. Black dots represent individual-level expression.
(B) Protein binding partners of HIPK1 as determined by StringDB. Green lines represent associations gleaned by text mining, purple lines represent results of direct binding experiments, blue lines represent associations curated from other databases, and brown lines represent associations determined through coexpression.
(C) Violin plots of PML normalized gene expression, measured as log2(counts per million), for healthy control subjects and patients with active disease and under treatment. Black dots represent individual-level expression.

expressed between individuals with active disease and HC subjects in the same direction, but to a lesser degree, as *HIPK1* (Figure 3C). The remaining binding partners of HIPK1 in the gene regulatory network were cross-referenced to the FactorBook transcription factor repository to determine which of the proteins acted as downstream transcription factors for HIPK1 signaling. Two factors were identified: p53 and GATA4. Transcription factor binding data, determined with ChIP-seq, were downloaded from FactorBook for both factors and run through BETA using the differential gene expression dataset. As is shown in Figures 4A–4C, both factors show a strong statistical enrichment of binding sites nearby up-regulated genes, as opposed to down-regulated or non-DEGs.

Based on the findings presented here, we propose the following mechanism. In healthy CD4+ T cells, under activated conditions, *HIPK1* plays a negative feedback role, acting to suppress GATA4 and TP53 and reduce their gene expression programs. In JIA, genetic variants in the vicinity of the rs6679677 index SNP result in the suppression of *HIPK1* expression, reducing the effectiveness of its regulatory role on GATA4 and TP53, prolonging a pro-inflammatory gene expression program that results in a hyper- and/or auto-inflammatory phenotype (Figure 5). This mechanism represents an ideal target for therapeutic intervention. Indeed, TP53 is a target for several drug modalities, such as small molecules and oligonucleotides, for the treatment of different oncological indications, demonstrating the pathway's potential.

Discussion

While the field of pediatric rheumatology has made significant advances in treating JIA in the past 20 years, significant challenges remain. For example, in a recent review using both Cincinnati Children's Hospital Medical Center and Children's Arthritis and Rheumatology Research Alliance data (from a total of 8,978 patients), Brunner et al.[32] found that more than half of the patients were treated with one or more biological agents and that >10% of the patients continued to have active disease despite having been treated with ≥2 biological agents. Indeed, among those patients treated with ≥2 biological agents, more than half continued to experience active disease. Furthermore, the emergence of validated tools to capture

Table 4. HIPK1 is involved in numerous critical processes

GO term	Description	FDR value
0030155[a]	regulation of cell adhesion	1.63E−03
0090287	regulation of cellular response to growth factor stimulus	8.76E−04
0006928	movement of cell or subcellular component	2.24E−03
0090092	regulation of transmembrane receptor protein serine/threonine kinase signaling pathway	5.73E−03
1903053	regulation of extracellular matrix organization	5.58E−03
0050794[a]	regulation of cellular process	5.93E−03
0090288	negative regulation of cellular response to growth factor stimulus	6.16E−03
0043087	regulation of GTPase activity	7.04E−03
0023051[a]	regulation of signaling	1.06E−02
0090101	negative regulation of transmembrane receptor protein serine/threonine kinase signaling pathway	1.03E−02
0016477	cell migration	1.26E−02
0010646[a]	regulation of cell communication	1.20E−02
0051056	regulation of small GTPase-mediated signal transduction	1.17E−02
0007010	cytoskeleton organization	1.32E−02
1903391	regulation of adherens junction organization	1.26E−02

Top 15 GO terms enriched among the JIA down-regulated genes.
[a]Terms that are associated with HIPK1.

patient-centered assessments of treatment outcomes[33] is likely to reveal additional challenges not immediately obvious to physicians but unacceptable from a parent's/patient's point of view.

A major impediment to developing improved, more precisely targeted therapies in JIA is our poor understanding of either the pathobiology of the disease or the biology of therapeutic response. With respect to the latter, we know that clinically derived descriptions of therapeutic response (active disease, inactive disease, and CRM) as established in the Wallace criteria[15] have objective correspondents in peripheral blood gene expression profiles.[16] Therapeutic response is associated with complex re-organization of peripheral blood gene expression networks,[17,34] but the most important elements of re-organization required to drive remission remain unknown. It is now recognized that remission is not typically associated with the normalization of peripheral blood gene expression profiles.[35] However, while transcriptome studies have provided some useful information about the complex biology of treatment response, they have, by themselves, not led to the identification of novel or unrecognized therapeutic targets.

In the current study, we used a combined genetics-genomics-transcriptomics approach to identify new therapeutic target candidates. The validity of our approach is suggested by the fact that we agnostically identified known therapeutic targets, albeit not at a statistically enriched level relative to OTG alone, such as cell surface integrins CD11a and CD11b. Indeed, the anti-CD11a monoclonal antibody (mAb) efalizumab was used to treat autoimmune diseases, including psoriasis and psoriatic arthritis, and was tested in rheumatoid arthritis (Table S2) before being withdrawn for safety issues. More recently, Schittenhelm et al. showed that altered expression patterns of CD11a and CD11b in dendritic cells were associated with inflammation regulation in adult rheumatoid arthritis.[36]

In the current study, drug tractability was an important criterion for the selection process, and *HIPK1* is notable for being a member of a druggable family. However, as a Ser/Thr kinase family member, the modality being referenced is a kinase inhibitor, which, if targeted against HIPK1 in the context of JIA, would be predicted to exasperate the problem. As such, HIPK1 itself may not represent a suitable drug candidate for JIA but rather an anchor point around which a search for a more suitable upstream regulator or downstream effector can take place.

The findings presented here regarding HIPK1's role in the pathogenesis of JIA have interesting therapeutic implications for other indications. Bei et al. recently showed that the inhibition of HIPK1 in cardiomyocytes was a novel therapeutic approach to treating hypertrophic cardiomyopathy (HTC).[37] Based on the data presented here, however, such a treatment would run the risk of inducing similar phenotypic consequences to JIA. Moreover, given that the rs6679677 SNP is associated with other indications beyond JIA, such as type 1 diabetes,[38] rheumatoid arthritis,[39] and systemic lupus erythematosus,[40] it is possible that HIPK1 inhibition in the context of treating HTC may result in immune-modulated adverse reactions beyond synovial joints in other tissues, such as the pancreas. To avoid such adverse events, it may prove necessary to deploy the HIPK1 inhibitors using an active targeting approach by attaching it to a mAb to form an antibody-drug conjugate (ADC) or to enclose it in a liposome and attach mAbs to the liposome surface to create an antibody-conjugated liposome (ACL).

There are several limitations to this study. The first is that the wet lab and procedures here were performed exclusively with CD4+ T cells. Although these cells are known to be an important feature of the immunopathology of JIA,[10] there is compelling evidence that both neutrophils[41,42] and monocytes[43,44] are involved in both genetic risk and disease biology. Thus, there are small but significant differences in 3D chromatin architecture across cells and cell lines that may highlight additional target candidates. A second limitation is the fact that our method, like that of Fang et al.,[7] uses the genome-wide association study (GWAS)-identified SNPs as a starting point for surveying genetic architecture. Because of the phenomenon of linkage disequilibrium, the SNPs that tag risk loci are not necessarily those that drive disease risk. It is probable that knowing the actual risk-driving variants and

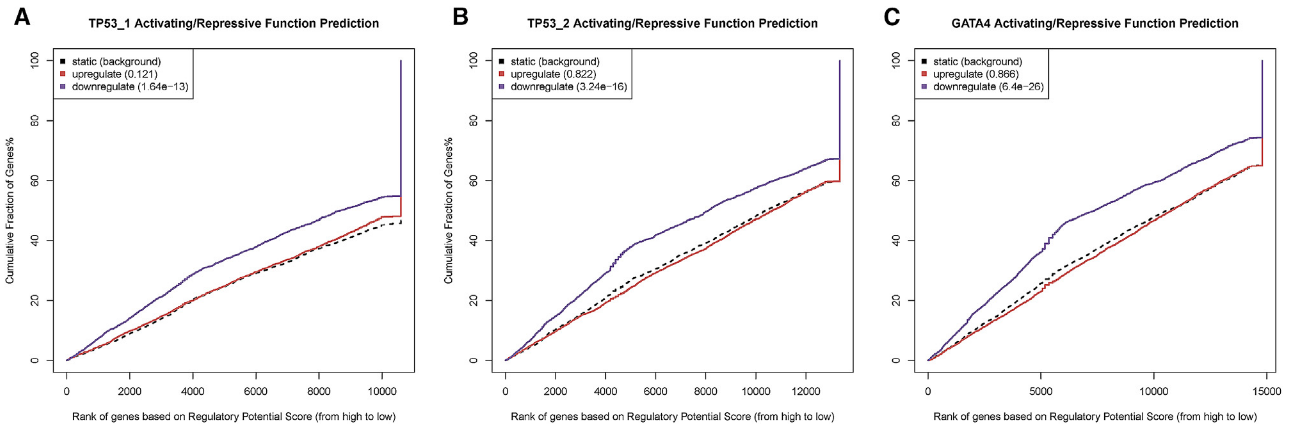

Figure 4. Regulatory potential analyses

(A) Regulatory potential of TP53 ChIP-seq binding sites identified in A549 cells for differentially expressed genes in T cells of patients with JIA.

(B) Regulatory potential of TP53 ChIP-seq binding sites identified in HepG2 cells for differentially expressed genes in T cells of patients with JIA.

(C) Regulatory potential of GATA4 ChIP-seq binding sites identified in HepG2 cells for differentially expressed genes in T cells of patients with JIA.

BETA generated the cumulative distribution function of the gene groups and the results of a one-tailed Kolmogorov-Smirnov test to determine whether the up-regulated and down-regulated groups differ significantly from the non-differential group. The dotted line represents the background, the genes that are not differentially expressed, whereas the red and the blue lines represent the genes up-regulated and down-regulated, respectively. The y axis represents the proportion of genes in a category that are ranked at or better than the x axis value, which represents the rank on the basis of the regulatory potential score from high to low. The *p* value listed in the top left represents the significance of the UP or DOWN group relative to the NON group as determined by the Kolmogorov-Smirnov test.

using higher-resolution maps of 3D chromatin (e.g., using MicroC)[45,46] will allow the identification of additional targets and more robust precision medicine approaches to treating JIA.

In conclusion, we demonstrate the use of genetic, genomic, and transcriptomic data in CD4+ T cells to identify new therapeutic targets in JIA. Our approach, which identified genes within or downstream of the GATA4-p53 pathways as potential targets, also provides new insights into the pathobiology of JIA. The role of genetic variants in attenuating HIPK1-mediated inhibition of CD4+ T cell activation deserves further investigation.

Data and code availability

Data from RNA-seq, ATAC-seq, and ChIP-seq/HiChIP for CTCF in purified CD4+ T cells is available on the Gene Expression Omnibus under accession number GEO: GSE164215. FactorBook ChIP-seq datasets are available under accessions ENCFF849LSO, ENCFF699UTZ, and ENCFF154VTV. Code is available upon request.

Acknowledgments

This work was supported by R01 AR078785 from NIH/NIAMS (J.N.J.) and an Innovative Research Grant from the Rheumatology Research Foundation.

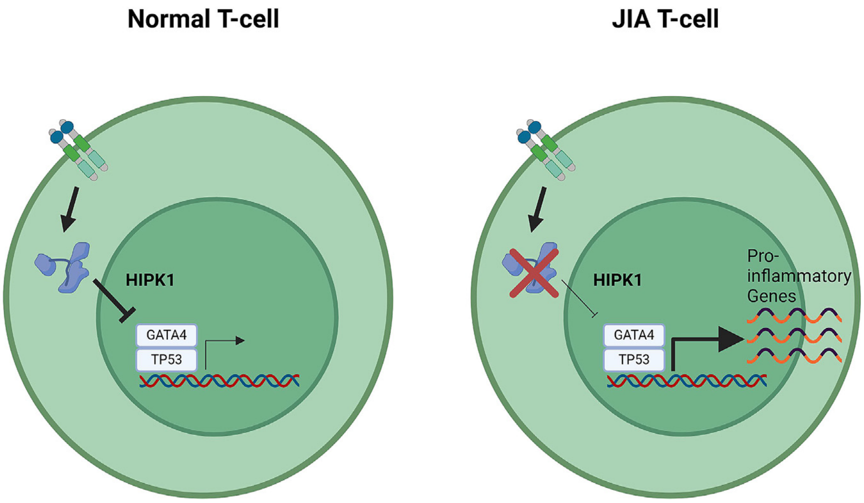

Figure 5. Model of HIPK1's role in the pathogenesis of JIA

In healthy CD4+ T cells, under activated conditions, *HIPK1* acts as a negative feedback, acting to suppress GATA4 and TP53 binding and reduce their gene expression programs. In JIA, genetic variants in the vicinity of the rs6679677 index SNP reduce *HIPK1* expression, lowering the effectiveness of its regulatory role on GATA4 and TP53 and prolonging a pro-inflammatory gene expression program that results in a hyper- and/or auto-inflammatory phenotype.

Declaration of interests

The authors declare no competing interests.

Supplemental information

Supplemental information can be found online at https://doi.org/10.1016/j.xhgg.2025.100424.

Web resources

FactorBook, https://www.factorbook.org/downloads
GEO, https://www.ncbi.nlm.nih.gov/geo/

Received: October 29, 2024
Accepted: March 10, 2025

References

1. Gortmaker, S.L., and Sappenfield, W. (1984). Chronic childhood disorders: prevalence and impact. Ped. Clin. North Am. *31*, 3–18.
2. Singsen, B.H. (1990). Rheumatic diseases of childhood. Rheum. Dis. Clin. North Am. *16*, 581–599.
3. Vanoni, F., Minoia, F., and Malattia, C. (2017). Biologics in juvenile idiopathic arthritis: a narrative review. Eur. J. Pediatr. *176*, 1147–1153. https://doi.org/10.1007/s00431-017-2960-6.
4. Chédeville, G., McGuire, K., Cabral, D.A., Shiff, N.J., Rumsey, D.G., Proulx-Gauthier, J.P., Schmeling, H., Berard, R.A., Batthish, M., Soon, G., et al. (2022). on Health-Related Quality of Life in Children With Juvenile Idiopathic Arthritis. Arthritis Care Res. *74*, 1567–1574. https://doi.org/10.1002/acr.24610.
5. Foell, D., Wulffraat, N., Wedderburn, L.R., Wittkowski, H., Frosch, M., Gerss, J., Stanevicha, V., Mihaylova, D., Ferriani, V., Tsakalidou, F.K., et al. (2010). Methotrexate withdrawal at 6 vs 12 months in juvenile idiopathic arthritis in remission: a randomized clinical trial. JAMA *303*, 1266–1273.
6. Packham, J.C., and Hall, M.A. (2002). Long-term follow-up of 246 adults with juvenile idiopathic arthritis: functional outcome. Rheumatology *41*, 1428–1435.
7. Fang, H., The ULTRA-DD Consortium, De Wolf, H., Knezevic, B., Burnham, K.L., Osgood, J., Sannit, A., Lara, A.L., Kasela, S., and De Cesco, S. (2019). A genetics-led approach defines the drug target landscape of 30 immune-related traits. Nature Genet. *51*, 1082–1091.
8. Owen, K.A., Price, A., Ainsworth, H., Aidukaitis, H.A., Bachali, P., Catalina, M.D., Dittman, J.M., Howard, T.D., Kingsmore, K.M., Labonte, A.C., et al. (2020). Analysis of trans-ancestral SLE risk loci identifies unique biologic networks and drug targets in African and European ancestries. Am. J. Human Genet. *107*, 1–18. https://doi.org/10.1016/j.ajhg.2020.09.000.
9. GTEx Consortium (2020). The GTEx Consortium atlas of genetic regulatory effects across human tissues. Science *369*, 1318–1330. https://doi.org/10.1126/science.aaz1776.
10. Tarbell, E., Jiang, K., Hennon, T.R., Holmes, L., Williams, S., Fu, Y., Gaffney, P.M., Liu, T., and Jarvis, J.N. (2021). CD4+ T cells from children with active juvenile idiopathic arthritis show altered chromatin features associated with transcriptional abnormalities. Sci. Rep. *11*, 4011. https://doi.org/10.1038/s41598-021-82989-5.
11. Grom, A.A., and Hirsch, R. (2000). T-cell and T-cell receptor abnormalities in the immunopathogenesis of juvenile rheumatoid arthritis. Curr. Op. Rheumatol. *12*, 420–424.
12. Prelog, M., Schwarzenbrunner, N., Sailer-Hoeck, M., Kern, H., Koppelstaetter, C., Wurzner, R., Zimmerhackl, L.B., and Brunner, J. (2008). Indications for a disturbed peripheral T-cell homeostasis in juvenile idiopathic arthritis (JIA): absent expansion of CD28 T-cells and no decrease of naive T-cells in cytomegalovirus-positive patients with JIA. J. Rheumatol. *35*, 520–527.
13. Ellis, J.A., Munro, J.E., Chavez, R.A., Gordon, L., Joo, J.E., Akikusa, J.D., Allen, R.C., Ponsonby, A.L., Craig, J.M., and Saffery, R. (2012). Genome-scale case-control analysis of CD4+ T-cell DNA methylation in juvenile idiopathic arthritis reveals potential targets involved in disease. Clin. Epigenet. *4*, 20. https://doi.org/10.1186/1868-7083-4-20.
14. Petty, R.E., Southwood, T.R., Manners, P., Baum, J., Glass, D.N., Goldenberg, J., He, X., Maldonado-Cocco, J., Orozco-Alcala, J., Prieur, A.M., et al. (2004). International League of Associations for Rheumatology classification of juvenile idiopathic arthritis: second revision, Edmonton, 2001. J. Rheumatol. *31*, 390–392.
15. Wallace, C.A., Huang, B., Bandeira, M., Ravelli, A., and Giannini, E.H. (2005). Patterns of clinical remission in select categories of juvenile idiopathic arthritis. Arthritis Rheum. *52*, 3554–3562. https://doi.org/10.1002/art.21389.
16. Knowlton, N., Jiang, K., Frank, M.B., Aggarwal, A., Wallace, C., McKee, R., Chaser, B., Tung, C., Smith, L., Chen, Y., et al. (2009). The meaning of clinical remission in polyarticular juvenile idiopathic arthritis: gene expression profiling in peripheral blood mononuclear cells identifies distinct disease states. Arthritis Rheum. *60*, 892–900. https://doi.org/10.1002/art.24298.
17. Du, N., Jiang, K., Sawle, A.D., Frank, M.B., Wallace, C.A., Zhang, A., and Jarvis, J.N. (2015). Dynamic tracking of functional gene modules in treated juvenile idiopathic arthritis. Genome Med. *7*, 109. https://doi.org/10.1186/s13073-015-0227-2.
18. Szklarczyk, D., Kirsch, R., Koutrouli, M., Nastou, K., Mehryary, F., Hachilif, R., Gable, A.L., Fang, T., Doncheva, N.T., Pyysalo, S., et al. (2023). The STRING database in 2023: protein–protein association networks and functional enrichment analyses for any sequenced genome of interest. Nucleic Acids Res. *51*, D638–D646.
19. Wang, J., Zhuang, J., Iyer, S., Lin, X., Whitfield, T.W., Greven, M.C., Pierce, B.G., Dong, X., Kundaje, A., Cheng, Y., et al. (2012). Sequence features and chromatin structure around the genomic regions bound by 119 human transcription factors. Genome Res. *22*, 1798–1812. https://doi.org/10.1101/gr.139105.112.
20. The Galaxy Community (2022). The Galaxy platform for accessible, reproducible and collaborative biomedical analyses: 2022 update. Nucleic Acids Res. *50*, W345–W351. https://doi.org/10.1093/nar/gkac247.
21. Leinonen, R., Sugawara, H., Shumway, M.; and International Nucleotide Sequence Database Collaboration (2011). The Sequence Read Archive. Nucleic Acids Res. *39*, D19–D21. https://doi.org/10.1093/nar/gkq1019.
22. NCBI. (n.d.). sra-tools. In GitHub repository. GitHub. https://github.com/ncbi/sra-tools.
23. Dobin, A., Davis, C.A., Schlesinger, F., Drenkow, J., Zaleski, C., Jha, S., Batut, P., Chaisson, M., and Gingeras, T.R. (2013).

STAR: ultrafast universal RNA-seq aligner. Bioinformatics *29*, 15–21. https://doi.org/10.1093/bioinformatics/bts635.
24. Harrow, J., Frankish, A., Gonzalez, J.M., Tapanari, E., Diekhans, M., Kokocinski, F., Aken, B.L., Barrell, D., Zadissa, A., Searle, S., et al. (2012). GENCODE: the reference human genome annotation for The ENCODE Project. Genome Res. *22*, 1760–1774. https://doi.org/10.1101/gr.135350.111.
25. Robinson, M.D., McCarthy, D.J., and Smyth, G.K. (2010). edgeR: a Bioconductor package for differential expression analysis of digital gene expression data. Bioinformatics *26*, 139–140. https://doi.org/10.1093/bioinformatics/btp616.
26. Eden, E., Navon, R., Steinfeld, I., Lipson, D., and Yakhini, Z. (2009). GOrilla: A Tool For Discovery And Visualization of Enriched GO Terms in Ranked Gene Lists",. BMC Bioinf. *10*, 48.
27. Wang, S., Sun, H., Ma, J., Zang, C., Wang, C., Wang, J., Tang, Q., Meyer, C.A., Zhang, Y., and Liu, X.S. (2013). Target analysis by integration of transcriptome and ChIP-seq data with BETA. Nat. Protoc. *8*, 2502–2515. https://doi.org/10.1038/nprot.2013.150.
28. Ghoussaini, M., Mountjoy, E., Carmona, M., Peat, G., Schmidt, E.M., Hercules, A., Fumis, L., Miranda, A., Carvalho-Silva, D., Buniello, A., et al. (2021). Open Targets Genetics: systematic identification of trait-associated genes using large-scale genetics and functional genomics. Nucleic Acids Res. *49*, D1311–D1320. https://doi.org/10.1093/nar/gkaa840.
29. DeNardo, D.G., Galkin, A., Dupont, J., Zhou, L., and Bendell, J. (2021). GB1275, a first-in-class CD11b modulator: rationale for immunotherapeutic combinations in solid tumors. J. Immunother. Cancer *9*, e003005. https://doi.org/10.1136/jitc-2021-003005.
30. Finan, C., Gaulton, A., Kruger, F.A., Lumbers, R.T., Shah, T., Engmann, J., Galver, L., Kelley, R., Karlsson, A., Santos, R., et al. (2017). The druggable genome and support for target identification and validation in drug development. Sci. Transl. Med. *9*, eaag1166. https://doi.org/10.1126/scitranslmed.aag1166.
31. Chen, S., Francioli, L.C., Goodrich, J.K., Collins, R.L., Kanai, M., Wang, Q., Alföldi, J., Watts, N.A., Vittal, C., Gauthier, L.D., et al. (2024). A genomic mutational constraint map using variation in 76,156 human genomes. Nature *625*, 92–100.
32. Brunner, H.I., Schanberg, L.E., Kimura, Y., Dennos, A., Co, D.O., Colbert, R.A., Fuhlbrigge, R.C., Goldmuntz, E., Kingsbury, D.J., Patty-Resk, C., et al. (2020). New Medications Are Needed for Children With Juvenile Idiopathic Arthritis. Arthritis Rheumatol. *72*, 1945–1951. https://doi.org/10.1002/art.41390.
33. Balay-Dustrude, E., and Shenoi, S. (2023). Current Validated Clinical and Patient Reported Disease Outcome Measures in Juvenile Idiopathic Arthritis. Open Access Rheumatol. *15*, 189–206. https://doi.org/10.2147/OARRR.S261773.
34. Hu, Z., Jiang, K., Frank, M.B., Chen, Y., and Jarvis, J.N. (2018). Modeling Transcriptional Rewiring in Neutrophils Through the Course of Treated Juvenile Idiopathic Arthritis. Sci. Rep. *8*, 7805. https://doi.org/10.1038/s41598-018-26163-4.
35. Jiang, K., Frank, M., Chen, Y., Osban, J., and Jarvis, J.N. (2013). Genomic characterization of remission in juvenile idiopathic arthritis. Arthritis Res. Ther. *15*, R100. https://doi.org/10.1186/ar4280.
36. Schittenhelm, L., Robertson, J., Pratt, A.G., Hilkens, C.M., and Morrison, V.L. (2021). Dendritic cell integrin expression patterns regulate inflammation in the rheumatoid arthritis joint. Rheumatology *60*, 1533–1542. https://doi.org/10.1093/rheumatology/keaa686.
37. Bei, Y., Zhu, Y., Wei, M., Yin, M., Li, L., Chen, C., Huang, Z., Liang, X., Gao, J., Yao, J., et al. (2023). HIPK1 Inhibition Protects against Pathological Cardiac Hypertrophy by Inhibiting the CREB-C/EBPβ Axis. Adv. Sci. *10*, e2300585. https://doi.org/10.1002/advs.202300585.
38. Chiou, J., Geusz, R.J., Okino, M.L., Han, J.Y., Miller, M., Melton, R., Beebe, E., Benaglio, P., Huang, S., Korgaonkar, K., et al. (2021). Interpreting type 1 diabetes risk with genetics and single-cell epigenomics. Nature *594*, 398–402. https://doi.org/10.1038/s41586-021-03552-w.
39. Stahl, E.A., Raychaudhuri, S., Remmers, E.F., Xie, G., Eyre, S., Thomson, B.P., Li, Y., Kurreeman, F.A.S., Zhernakova, A., Hinks, A., et al. (2010). Genome-wide association study meta-analysis identifies seven new rheumatoid arthritis risk loci. Nat. Genet. *42*, 508–514. https://doi.org/10.1038/ng.582.
40. Bentham, J., Morris, D.L., Graham, D.S.C., Pinder, C.L., Tombleson, P., Behrens, T.W., Martín, J., Fairfax, B.P., Knight, J.C., Chen, L., et al. (2015). Genetic association analyses implicate aberrant regulation of innate and adaptive immunity genes in the pathogenesis of systemic lupus erythematosus. Nat. Genet. *47*, 1457–1464. https://doi.org/10.1038/ng.3434.
41. Jarvis, J.N., Petty, H.R., Tang, Y., Frank, M.B., Tessier, P.A., Dozmorov, I., Jiang, K., Kindzelski, A., Chen, Y., Cadwell, C., et al. (2006). Evidence for chronic, peripheral activation of neutrophils in polyarticular juvenile rheumatoid arthritis. Arthritis Res. Ther. *8*, R154.
42. Jarvis, J.N., Jiang, K., Frank, M.B., Knowlton, N., Aggarwal, A., Wallace, C.A., McKee, R., Chaser, B., Tung, C., Smith, L.B., et al. (2009). Gene expression profiling in neutrophils of children with polyarticular juvenile idiopathic arthritis. Arthritis Rheum. *60*, 1488–1495.
43. Haley, E.K., Barshad, G., He, A., Rice, E., Sudman, M., Thompson, S.D., Crinzi, E.A., Jiang, K., Danko, C.G., and Jarvis, J.N. (2024). Using functional genomic data in monocytes/macrophages and genotyping to nominate disease-driving single nucleotide polymorphisms and target genes in juvenile idiopathic arthritis. Preprint at bioRxiv. https://doi.org/10.1101/2024.08.19.608312.
44. Crinzi, E.A., Haley, E.K., Poppenberg, K.E., Jiang, K., Tutino, V.M., and Jarvis, J.N. (2022). Analysis of chromatin data supports a role for CD14+ monocytes in mediating genetic risk for juvenile idiopathic arthritis. Front. Immunol. *13*, 913555. https://doi.org/10.3389/fimmu.2022.913555.
45. Hsieh, T.H.S., Weiner, A., Lajoie, B., Dekker, J., Friedman, N., and Rando, O.J. (2015). Mapping Nucleosome Resolution Chromosome Folding in Yeast by Micro-C. Cell *162*, 108–119. https://doi.org/10.1016/j.cell.2015.05.048.
46. Goel, V.Y., Huseyin, M.K., and Hansen, A.S. (2023). Region Capture Micro-C reveals coalescence of enhancers and promoters into nested microcompartments. Nat. Genet. *55*, 1048–1056. https://doi.org/10.1038/s41588-023-01391-1.

HGGA, Volume *6*

Supplemental information

Using genetics, genomics, and transcriptomics to identify therapeutic targets in juvenile idiopathic arthritis

Evan Tarbell and James N. Jarvis

Supplemental Table 2. Clinical Trials of putative gene targets

ClinicalTrials.gov ID
NCT00034203
NCT00276250
NCT00287118
NCT00669214
NCT00312026
NCT00302445
NCT00096980
NCT00336973
NCT00249808
NCT00737763
NCT00501709
NCT00051662
NCT00000278
NCT02101008
NCT03950830
NCT00312819
NCT00435435
NCT02735577

Clinical trial identification numbers for drugs against one of the putative target genes for JIA.
